# Analyzing the antagonistic potential of the lichen microbiome against pathogens by bridging metagenomic with culture studies

**DOI:** 10.3389/fmicb.2015.00620

**Published:** 2015-06-22

**Authors:** Tomislav Cernava, Henry Müller, Ines A. Aschenbrenner, Martin Grube, Gabriele Berg

**Affiliations:** ^1^Institute of Environmental Biotechnology, Graz University of TechnologyGraz, Austria; ^2^Institute of Plant Sciences, University of GrazGraz, Austria

**Keywords:** lichen, antagonistic bacteria, plant growth promotion, *Stenotrophomonas*, spermidine

## Abstract

Naturally occurring antagonists toward pathogens play an important role to avoid pathogen outbreaks in ecosystems, and they can be applied as biocontrol agents for crops. Lichens present long-living symbiotic systems continuously exposed to pathogens. To analyze the antagonistic potential in lichens, we studied the bacterial community active against model bacteria and fungi by an integrative approach combining isolate screening, omics techniques, and high resolution mass spectrometry. The highly diverse microbiome of the lung lichen [*Lobaria pulmonaria* (L.) Hoffm.] included an abundant antagonistic community dominated by *Stenotrophomonas, Pseudomonas,* and *Burkholderia.* While antagonists represent 24.5% of the isolates, they were identified with only 7% in the metagenome; which means that they were overrepresented in the culturable fraction. Isolates of the dominant antagonistic genus *Stenotrophomonas* produced spermidine as main bioactive component. Moreover, spermidine-related genes, especially for the transport, were identified in the metagenome. The majority of hits identified belonged to *Alphaproteobacteria*, while *Stenotrophomonas*-specific spermidine synthases were not present in the dataset. Evidence for plant growth promoting effects was found for lichen-associated strains of *Stenotrophomonas.* Linking of metagenomic and culture data was possible but showed partly contradictory results, which required a comparative assessment. However, we have shown that lichens are important reservoirs for antagonistic bacteria, which open broad possibilities for biotechnological applications.

## Introduction

Plant pathogens and the diseases they cause are major threats to humanity. Each year we globally lose over one third of the total harvest to bacterial and fungal pathogens. The past two decades have seen an increasing number of virulent infectious diseases in plants (Fisher et al., 2012), and human activity is intensifying pathogen dispersal as well as reducing diversity in agricultural systems (Schmid et al., 2011). However, microbial diversity is a key factor in avoiding pathogen outbreaks (Mendes et al., 2012; van Elsas et al., 2012). Therefore, biocontrol of plant pathogens is a promising solution to control plant pathogens (Berendsen et al., 2012; Berg et al., 2013; Berg, 2015) because it was also shown that it enhances general microbial diversity (Erlacher et al., 2015a). Naturally occurring antagonists toward plant pathogens play an important role for biocontrol approaches. In natural ecosystems, which often contain a high proportion of antagonistic microorganisms, such antagonists potentially function in stabilizing the community, but might also protect the community against pathogen outbreaks (Opelt et al., 2007; Zachow et al., 2008; Grube et al., 2015). However, the ecology of naturally occurring antagonistic microorganisms is only partly understood and not yet exploited.

Lichens, which are classic examples of self-sustained symbioses, are interesting models for antagonism studies because within these mini-ecosystems the cooperation between microbial partners facilitates stability and longevity under extreme ecological conditions although they are often attacked by allochthonous bacteria and fungi (Lawrey and Diederich, 2003; Bates et al., 2011; Mushegian et al., 2011). While the lichen-specific structure is provided by fungal symbionts, which also is the naming component of the symbiosis, green algae, and/or cyanobacteria are incorporated into specific layers or compartments and contribute with photosynthetically fixed carbohydrates to the symbiosis (Nash, 2008). Lichen-associated bacteria were only recently shown to be highly diverse and omics approaches have indicated that they are functional contributors to robustness of the lichen holobiome (Grube et al., 2009, 2015). The intricate association of members of different organismal kingdoms in well-delimited and long-living symbiotic structures – as symbiotic hotspots of terrestrial life – highlights lichens as a veritable treasure chest for interorganismal communication, regulation, and bioactivity in general (Boustie and Grube, 2005; Boustie et al., 2011). Conditioned by the slow growth of many lichens and difficulties in culturing the symbionts, biotechnological exploitation of lichens was lagging behind other natural resources. With the advent of modern technologies, however, the secondary metabolism and antagonistic potentials in lichens receive new impulses, and this will particularly apply to culturable bacterial partners. Although, lichens are equipped with various secondary compounds with antagonistic effects (Oksanen, 2006; Lawrey, 2009; Boustie et al., 2011), we hypothesize that only a diverse protective microbiome can efficiently maintain stability over longer periods to prevent pathogen attacks.

The objective of this study was to analyze the antagonistic potential of the lichen microbiome against model pathogens by a novel approach bridging metagenomic with culture techniques. Model pathogens associated with human, lichen and plant diseases were accessed to screen for a broad spectrum of antagonistic activity. Furthermore, we utilized the lung lichen *Lobaria pulmonaria* (L.) Hoffm., which is one of the fastest growing leaf-like lichens (MacDonald and Coxson, 2013) and used as indicator species of undisturbed forests and air pollution (Rose, 1976; Scheidegger, 1995). We also characterized the most active as well as the most abundant lichen-associated antagonists *Stenotrophomonas,* which were already identified as versatile antagonists from plant origin (Ryan et al., 2009; Alavi et al., 2014; Berg and Martinez, 2015). Beneficial *Stenotrophomonas* strains produced osmoprotectans and spermidine in response to eukaryotic hosts (Alavi et al., 2013). In our study we applied multidisciplinary techniques to link metagenomic data with those obtained from bacterial cultures. Moreover, we could show that lichens are important reservoirs for antagonistic bacteria, which can also be used for biological control approaches to protect plants against biotic and abiotic stress.

## Materials and Methods

### Sampling Strategy and Isolation of Lichen-Associated Bacteria

Lichen thalli of *L. pulmonaria* were sampled from three different locations in Austria (Tamischbachgraben, N47°32′40^′′^, E14°37′35^′′^, Johnsbach, N47°38′07^′′^, E14°44′45^′′^, and St. Oswald ob Eibiswald, N46°44′ 50^′′^, E15° 04′ 26^′′^) after visual inspection to avoid contamination by lichenicolous fungi and other organisms. Five separate lichen thalli were sampled from each sampling site. The samples were stored on dry ice and were, shortly after, ground with mortar and pestle. A homogenate was prepared using sterile 0.85% NaCl in a 1:10 (w/v) ratio, together with a lab stomacher (BagMixer; Interscience, St Nom, France). Diluted fractions were plated on R2A agar (Carl Roth, Karlsruhe, Germany), R2A agar with 25 μg ml^-1^ cycloheximide, starch casein agar (SCA; Küster and Williams, 1964) and ISP2 agar (Shirling and Gottlieb, 1966). Bacterial colonies were randomly picked within 5 days of incubation at room temperature (RT) and a total of 388 isolates was obtained. The isolates were stored in glycerol stocks at -70°C prior to cultivation-based experiments.

### Screening of Isolates for *In Vitro* Antagonistic Activity Toward Particular Bacteria and Fungi

Dual-culture experiments were carried out as confrontation assays, using different media and target organisms according to Berg et al. (2002) and Opelt et al. (2007). Lichen-associated isolates were spotted on solid media pre-inoculated with *Escherichia coli* XL1 and *Staphylococcus aureus* ATCC 25923 and assessed for inhibition zones after 4 days of incubation at 30°C. Antagonistic activity against the fungus *Botrytis cinerea* Pers. (in-house culture collection) was tested by dual culture on Waksman agar (WA), according to Berg et al. (2002) and assessed after 5–7 days incubation at 20°C. Cultures of the lichen-pathogenic fungus *Rhinocladiella* sp. (culture collection of Lucia Muggia; Institute of Plant Sciences, University of Graz) were homogenized and re-suspended in sterile 0.85% NaCl. In the following step, 50 μL aliquots from one batch were used to inoculate each well of 24-well plates which contained solid potato dextrose agar (PDA; Carl Roth, Karlsruhe, Germany). Subsequently, 100 μL culture filtrate obtained from each lichen-associated isolate was added to particular wells. After 3 weeks of incubation, the wells were checked for growth reduction. All experiments were conducted with three independent replicates.

### Amplicon Library Preparation and Co-Occurrence Analysis

Amplicon libraries obtained by Aschenbrenner et al. (2014) were used to extract distinct taxa for additional studies. The utilized 454-pyrosequencing data was obtained from lichen samples from the same sampling sites that were used for isolation of lichen-associated bacterial cultures. Out of the 454-amplicon dataset 15 thallus samples (five for each sampling site) were used for a co-occurrence analysis. Therefore OTUs (Operational Taxonomic Units) were clustered with UCLUST (Edgar, 2010) at 95% similarity (correlates with the taxonomic genus level). Mitochondrial, chloroplast, and *Nostoc* sequences were excluded as well as all OTUs with less than three sequences. Co-occurrence patterns were created with calculated Spearman correlations between taxa at family level (>0.6 and <-0.6; R environment version 3.1.2^1^). Only families, which showed a correlation to *Pseudomonadaceae, Xanthomonadaceae,* and *Burkholderiaceae* were considered for further analysis and visualized as network with Cytoscape (organic layout; version 3.2.1; Saito et al., 2012). Node size within the network reflects the sequence abundance of each taxon and nodes were colored according to phylum affiliation.

### 16S rRNA-Based Identification of Antagonistic Bacteria and Phylogenetic Analysis

Primer pair 27F/1492r was used to amplify specific 16S rRNA gene fragments from antagonistic bacterial cultures. Subsequent sequencing and BLASTn searches within the 16S ribosomal RNA sequence database (NCBI) were conducted for identification of antagonists. These sequences were later trimmed to the hypervariable V4 region to allow alignments with 454-pyrosequencing data of the same rRNA region. QIIME 1.6.0 (Quantitative Insights Into Microbial Ecology, Caporaso et al., 2010) and the implemented Greengenes database (DeSantis et al., 2006) was used to search for bacterial OTUs in the corresponding amplicon dataset (Aschenbrenner et al., 2014) that were assigned to the three most dominant proteobacterial genera within prior identified antagonists. OTUs which could not be assigned to any particular phylum or to species level within *Proteobacteria* were additionally analyzed with Seqmatch (RDP database) for taxonomic assignment. The representative sequences of these additionally identified OTUs were used for further phylogenetic analyses. The phylogenetic tree was constructed with V4-trimmed 16S rRNA sequences from antagonistic bacteria cultures and an amplicon subset. Sequences were aligned with MEGA6 (Tamura et al., 2013) and processed for bootstrapped neighbor-joining with PHYLIP work package v.3.695^2^. Confidence levels for the internal branches were assessed by bootstrap analysis with 100 re-samplings. FigTree v.1.4.0^3^ was used for annotation and final graphic visualization of the phylogenetic tree. All utilized 16S rRNA gene fragment sequences from isolate and amplicon sequencing were deposited at GenBank^4^ (accession numbers: KP739786–KP739797 and KR611621–KR611709).

### Metagenomic Mining for Specific Genes of Interest

All metagenome-based analyzes were carried out on the assembled dataset described in a previous study by Grube et al. (2015). CLUSTER CONTROL (Stocker et al., 2004) was used to search with the blastn algorithm for specific spermidine synthase matches (NCBI accession numbers: NC_010943.1, NC_011071.1, NC_015947.1, and NC_017671.1) within the dataset (368,424 contigs). MEGAN (v4.70.4) was used to retrieve taxonomic classification and relevant SEED functions.

### Metabolite Extraction from Bacterial Cultures Grown on Solid Medium

Bacterial cultures were washed from several densely colonized Nutrient Agar (NA; Sifin, Berlin, Germany) plates after 48 h incubation at 30°C and homogenized in 9 mL 0.85% NaCl solution. The homogenate was centrifuged for 20 min, 2,000 *g* at 4°C. This step was repeated two times to remove residual media from bacterial cells. The pellet was re-suspended in 2 mL ddH_2_O followed by centrifugation for 15 min, 18,000 *g* at 4°C. Precooled 90% methanol at -70°C was used for reproducible extraction and to avoid further degradation of metabolites. Subsequently, 1 mL was added to each pellet and the bacterial cells were mechanically disrupted with glass beads for 2 × 45 s at 6 m/s. Followed by a final centrifugation step for 15 min, 18,000 *g* at 4°C, 100 μl of each supernatant was collected and immediately placed in a deep freezer at -70°C until further analysis. Three independent biological replicates were prepared for each isolate.

### Preparation of Culture Supernatants for Spermidine Quantification

Bacterial cultures were used to prepare overnight cultures (ONC) in fluid Nutrient Broth II (NBII; Sifin, Berlin, Germany) medium. These ONCs were used to inoculate 50 mL NBII flasks, which were then incubated at 30°C, 120 rpm for 48 h. In the following step, 2 mL aliquots were taken from the cultures and centrifuged for 20 min, 18,000 *g* at 4°C. The supernatants were filtered with 0.25 μm filters and immediately placed in a deep freezer at -70°C until further analysis. Three independent biological replicates were prepared for each isolate.

### Quantification of Specific Bacterial Metabolites with High Resolution Mass Spectrometry

Samples were analyzed in nine biological/technical replicates with a combined HPLC-hybrid quadrupole-orbitrap mass spectrometer (Q Exactive; Thermo Scientific, Bremen, Germany). A Luna 5u NH2 100A 250 × 4.6 column (Phenomenex, Aschaffenburg, Germany) was used to separate different metabolites from the cell extracts. Formic acid (0.1%, v/v) in acetonitrile was used as solvent A and aqueous formic acid (0.1%, v/v) as solvent B. Starting conditions for the gradient elution were 10% A and 90% B. The conditions were gradually changed to 80% A and 20% B within 15 min. This step was followed by 5 min at 10% A and 90% B for readjustment to initial conditions. The eluent flow was maintained at 0.8 mL/min together with a column temperature of 25°C. Sample analysis was carried out with negative ion ESI detection. ESI conditions were set to 3.2 kV spray voltage and 350°C capillary temperature. Scans were recorded in the range 100.0–300.0 m/z with the AGC target set to 500,000 and maximal accumulation time of 200 ms. The resolution was adjusted to 200,000. Altering full MS-SIM and targeted MS^2^ cycles were employed and a specific inclusion mass of 146.16517 amu was selected. Standard calibration was obtained with 0, 0.02, 0.03, 0.04, 0.05, 0.1, and 0.2 μM spermidine standard (Duchefa Biochemie, Haarlem, The Netherlands) diluted in 0.2 mM HCL.

### Plant Growth Experiments with *Stenotrophomonas*-Primed Seeds

Overnight cultures with selected *Stenotrophomonas* isolates were used to inoculate main cultures in fluid NBII. In addition to the lichen-associated isolates, a plant-associated isolate *Stenotrophomonas rhizophila* P69 (Minkwitz and Berg, 2001; Wolf et al., 2002) was also utilized for comparisons. After 2 h of growth at 30°C and 120 rpm the fluid cultures were diluted to 5 × 10^6^–1 × 10^7^ cells per mL in sterile 0.85% NaCl solution. Tomato (*Solanum lycopersicum* L. cv. Kremser Perle; Austrosaat, Graz, Austria) seeds were surface sterilized with 4% NaHClO for 10 min followed by drying at RT. The sterilized seeds were put into the respective bacterial suspensions and incubated for 4 h at 120 rpm and RT. The control samples were put in 0.85% NaCl solution without bacteria. Ground and homogenized seeds from each inoculum were plated on NA to test priming efficiency after the incubation time. The remaining seeds were planted in sterile soil (150 g/tray) with vermiculite (1:3 ratio) and watered with 30 mL sterile H_2_O. Beside the control with non-primed seeds an additional control with P69-primed seeds was added. Therefore, 60 μL 5-sec-butyl-2,3-dimethylpyrazine (Sigma–Aldrich, Steinheim, Germany) was supplemented into 30 mL sterile H_2_O used for irrigation after planting. The closed trays were placed without further irrigation for 2 weeks into a greenhouse with 12 h day/night cycles and a constant temperature of 24°C. Root (*n* = 64) and stem (*n* = 66) lengths of the plantlets were assessed separately for all samples.

### Statistical Analysis

Statistical analyses were performed with SPSS v.20.0.0 (SPSS Inc, Chicago, IL, USA). Data were tested for normal distribution with the Kolmogorov–Smirnov test. Sets with normally distributed data were analyzed with univariate ANOVA and Duncan tests at *p* < 0.05. The *t*-test was employed for statistical analysis of data that was not normally distributed (*p* < 0.05 and *p* < 0.1).

## Results

### Antagonistic Bacteria within the *Lobaria* Microbiome

**FIGURE 1:**
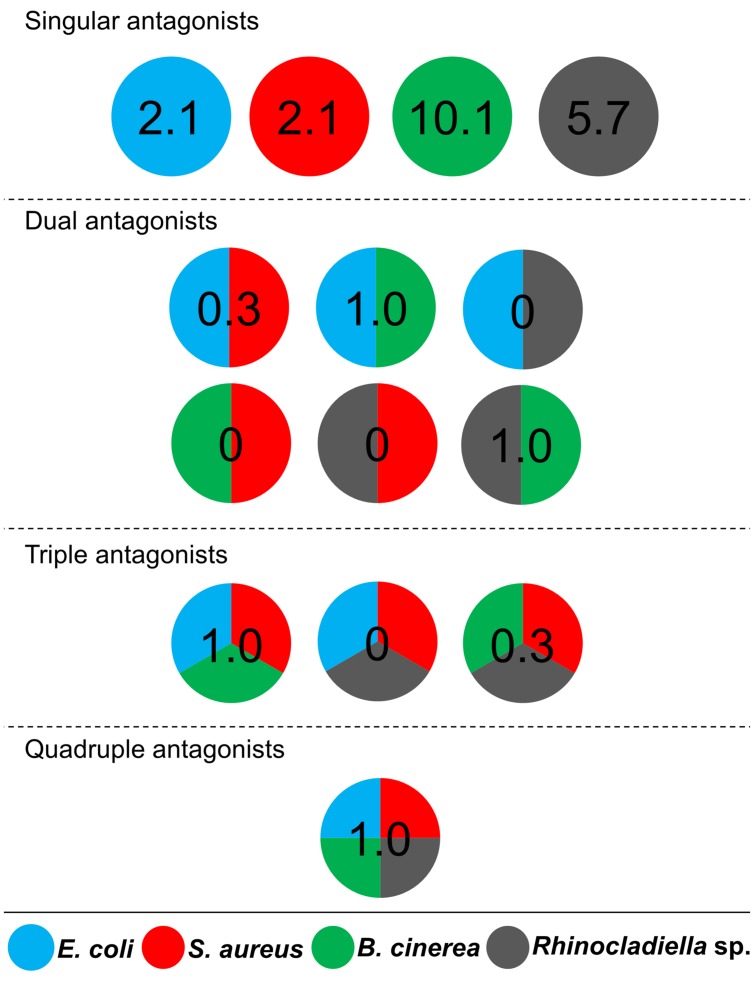
**Distribution of antagonistic bacteria isolated from multiple *Lobaria pulmonaria* thalli.** Dual-culture experiments with 388 bacterial cultures were used to identify antagonists against four different model pathogens. *Escherichia coli* and *Staphylococcus aureus* were used as models for human pathogens, *Botrytis cinerea* and *Rhinocladoniella* sp. were used as models for plant and lichen pathogens, respectively. Results are presented in a schematic illustration of antagonists, targeting a specific pathogen (indicated by relative numbers and different colors, respectively). Divided circles indicate particular combinations of pathogens, which were inhibited by an assigned percentage of *Lobaria*-associated isolates. Singular antagonists: *Lobaria*-associated isolates that inhibited only one model pathogen; Dual antagonists: isolates that inhibited two distinct model pathogens; Triple antagonists: isolates that inhibited three distinct model pathogens; Quadruple antagonists: isolates that inhibited four distinct model pathogens.

**FIGURE 2:**
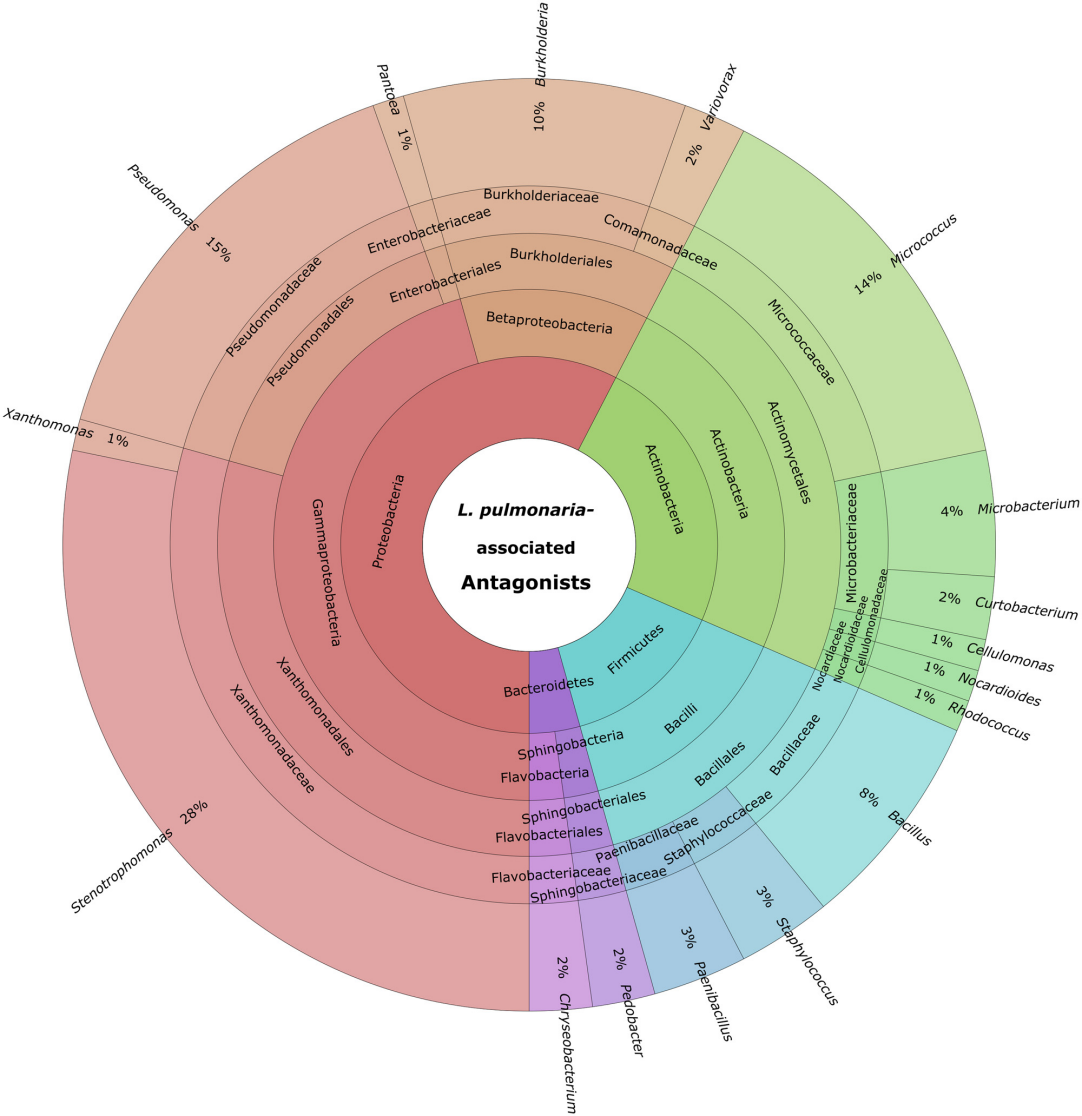
**Summary of identified antagonistic bacteria in a multi-level chart (http://sourceforge.net/p/krona).** The displayed taxa were shown to inhibit growth of one or more of the utilized model pathogens: *B. cinerea, E. coli, S. aureus,* and *Rhinocladionella* sp. The inner circles represent higher taxonomic ranks, while more detailed taxonomic ranks (up to genus level) are presented in the outer circles.

Cultivable bacteria, which have been isolated from 15 *L. pulmonaria* samples, were tested in dual-culture assays against the bacterial model pathogens *E. coli, S. aureus*, the plant pathogen *B. cinerea* and the lichenicolous fungus *Rhinocladoniella* sp. to determine the general antagonistic potential. In these experiments, lichen-associated antagonists were shown to primarily target lichen and plant pathogenic fungi (20.1% of all isolates) while a lower proportion was directed against bacterial model pathogens (7.7% of all isolates). A total of 95 isolates (24.5%) showed inhibition of growth of at least one of the model pathogens (**Figure 1**). Singular antagonists (active against only one target microorganism) were dominated by *Stenotrophomonas* spp. (31% of singular antagonists) followed by *Pseudomonas* spp. (19%) and *Burkholderia* spp. (12%; Supplementary Figure S1). Dual antagonists (active against two microorganisms) comprised nine isolates. *Stenotrophomonas* and *Micrococcus* were represented by three isolates and *Chryseobacterium, Microbacterium,* and *Paenibacillus* by only one isolate, respectively. Nine bacterial strains inhibited the growth of either three or four model pathogens simultaneously. These cultures were identified at genus level as *Bacillus* (five isolates), *Micrococcus* (one isolate), and *Paenibacillus* (three isolates). A complete taxonomic breakdown for all identified antagonists was visualized in **Figure 2**.

Comparison of the hypervariable V4 rRNA region from the most abundant proteobacterial antagonistic isolates: *Stenotrophomonas* spp., *Pseudomonas* spp., and *Burkholderia* spp. with filtered OTUs from an amplicon library constructed with *Lobaria* samples from the same sampling sites, revealed high homology of sequences within the same genus (Supplementary Figure S2). Specific branches (bootstrap values > 70%) were detected for amplicon- and isolate-based sequences. OTUs that were assigned to *Stenotrophomonas* sp., *Pseudomonas* sp., and *Burkholderia* sp. comprised, respectively, 0.06, 0.56, and 0.09% of all analyzed OTUs in the amplicon library. Interestingly three of the highly active antagonistic genera (*Bacillus, Micrococcus,* and *Paenibacillus*) were not substantially represented in the amplicon library.

**FIGURE 3:**
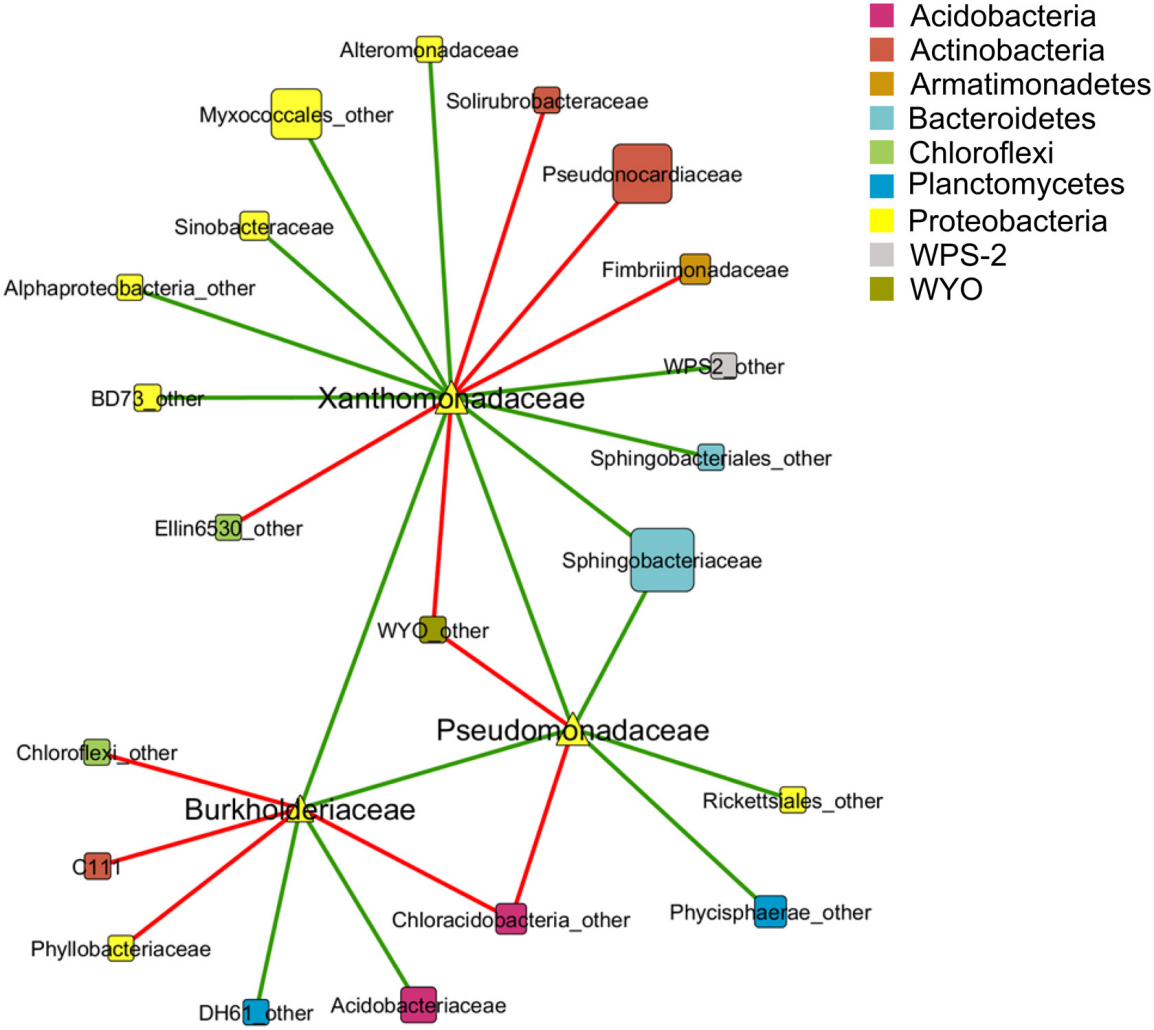
**Co-occurrence pattern between taxa of the lichen-associated microbiome.** Nodes represent taxa at family level which are colored by phylum affiliation. Node size reflects sequence abundance of each taxon. The edges represent Spearman correlations in green (positive correlation, *R* > 0.6) and red lines (negative correlation, *R* < -0.6).

A co-occurrence pattern between different taxa at family level was created with the calculated Spearman correlations based on 15 lichen thallus samples. In total, 24 correlations between the families *Pseudomonadaceae, Burkholderiaceae,* and *Xanthomonadaceae* to other taxa within the microbiome could be detected and were visualized as a co-occurrence network (**Figure 3**). Most correlations (10 out of 24) were found within the phylum *Proteobacteria* followed by *Actinobacteria* (3). The strongest positive correlations (Spearman correlation > ±0.7) showed *Xanthomonadaceae* with *Pseudomonadaceae* and *Alteromonadaceae*, all assigned to *Gammaproteobacteria*, whereas the strongest negative correlations were found between *Burkholderiaceae* (*Betaproteobacteria*) and *Phyllobacteriaceae* (*Alphaproteobacteria*) and a family within the class *Chloracidobacteria* (*Acidobacteria*) which was not further classified in the utilized database.

In addition, the abundance of antagonistic taxa was extracted from the *Lobaria* metagenome. The proportion of retrievable antagonistic genera was determined for *Stenotrophomonas* (0.22% of all bacteria within the metagenome), *Pseudomonas* (1.14%), *Burkholderia* (2.81%), *Xanthomonas* (0.43%), *Nocardiodes* (0.10%), *Rhodococcus* (0.18%), *Bacillus* (0.08%), and *Staphylococcus* (0.02%). The remaining antagonistic taxa could not be retrieved at genus level. However, they comprised at family level together 2.14% of all bacteria. Altogether, antagonistic taxa comprised 7.12% of the total bacterial community. Neither the genus *Cellulomonas* nor the family *Cellulomonadaceae* was present in the assembled metagenomic dataset.

### Spermidine Production *In Vitro* and Spermidine-Related Genes within the *Lobaria*-Associated Metagenome

Genes coding for spermidine synthases were analyzed from the *Lobaria* metagenome and taxonomically assigned. In addition, spermidine production and secretion was analyzed *in vitro*.

**FIGURE 4:**
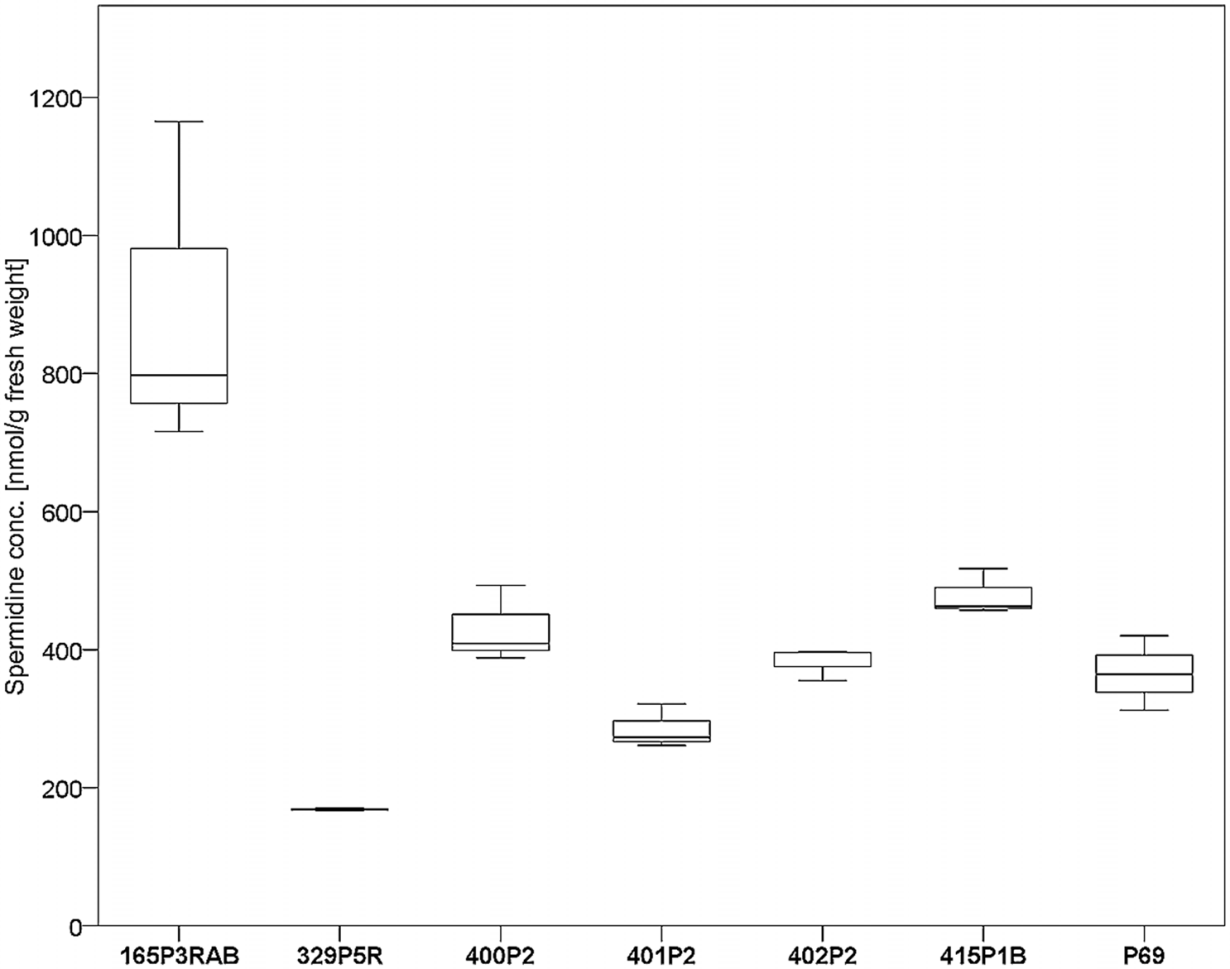
**Analysis of internal spermidine concentrations and lichen-associated *Stenotrophomonas* sp. isolates.** The isolates were cultivated on solid cultivation media followed by cell disruption and quantification of internal spermidine levels (*n* = 21). A total of six *Lobaria*-associated isolates were utilized together with one plant-associated isolate (P69) in a comparative approach. *Stenotrophomonas* sp. 165P3RAB was shown to contain the highest internal spermidine concentration (*p* < 0.1) compared to all other isolates, while isolate 329P5R had the lowest concentration (*p* < 0.01).

Seven antagonistic *Stenotrophomonas* sp. isolates were cultivated on solid agar plates and in liquid media prior to the extraction of spermidine. The detection limit for spermidine on the utilized instruments was determined to be <30 nM. Externalized spermidine levels detected in liquid cultivation media were in the range between 8.2 and 10.5 μmol/g fresh weight. Extracellular spermidine concentration differences between utilized *Stenotrophomonas* isolates were not statistically significant (Supplementary Figure S3). Conversely, the same isolates were shown to contain different internal spermidine concentrations after cultivation on solid media (**Figure 4**). The lowest internal spermidine concentration was found to be 168 nmol/g fresh weight, which was around fourfold lower than the highest observed concentration. Isolate 165P3RAB was found to contain significantly higher spermidine concentrations than all other isolates. In contrast, *Stenotrophomonas* isolate 329P5R contained the lowest spermidine concentrations.

For BLASTn searches, reference sequences for spermidine synthases from four different *Stenotrophomonas* strains were utilized. Additionally, SEED assignments were searched for related functions. *Stenotrophomonas*-specific contigs that contain known spermidine synthases were not found in the utilized metagenome, while three other bacteria-derived contigs were present. Two spermidine synthase contigs that were retrieved with SEED-based analysis were assigned to *Proteobacteria*. One contig was assigned to *Burkholderiaceae* and the other contig to *Acetobacteraceae*. A third contig could not be assigned to any taxon. Conversely, spermidine putrescine transporter permeases were more abundant in the metagenome. A total of 50 contigs were assigned to this specific transporter protein (Supplementary Figure S4). More than a half of these contigs were assigned to bacteria (58%), while 42% remained unassigned to a specific kingdom. The hits were predominantly associated with *Proteobacteria* (52%) and more specifically to *Rhizobiales* (28%).

### *Stenotrophomonas* Treatments Increased Plant Growth of Tomato Under Greenhouse Conditions

**FIGURE 5:**
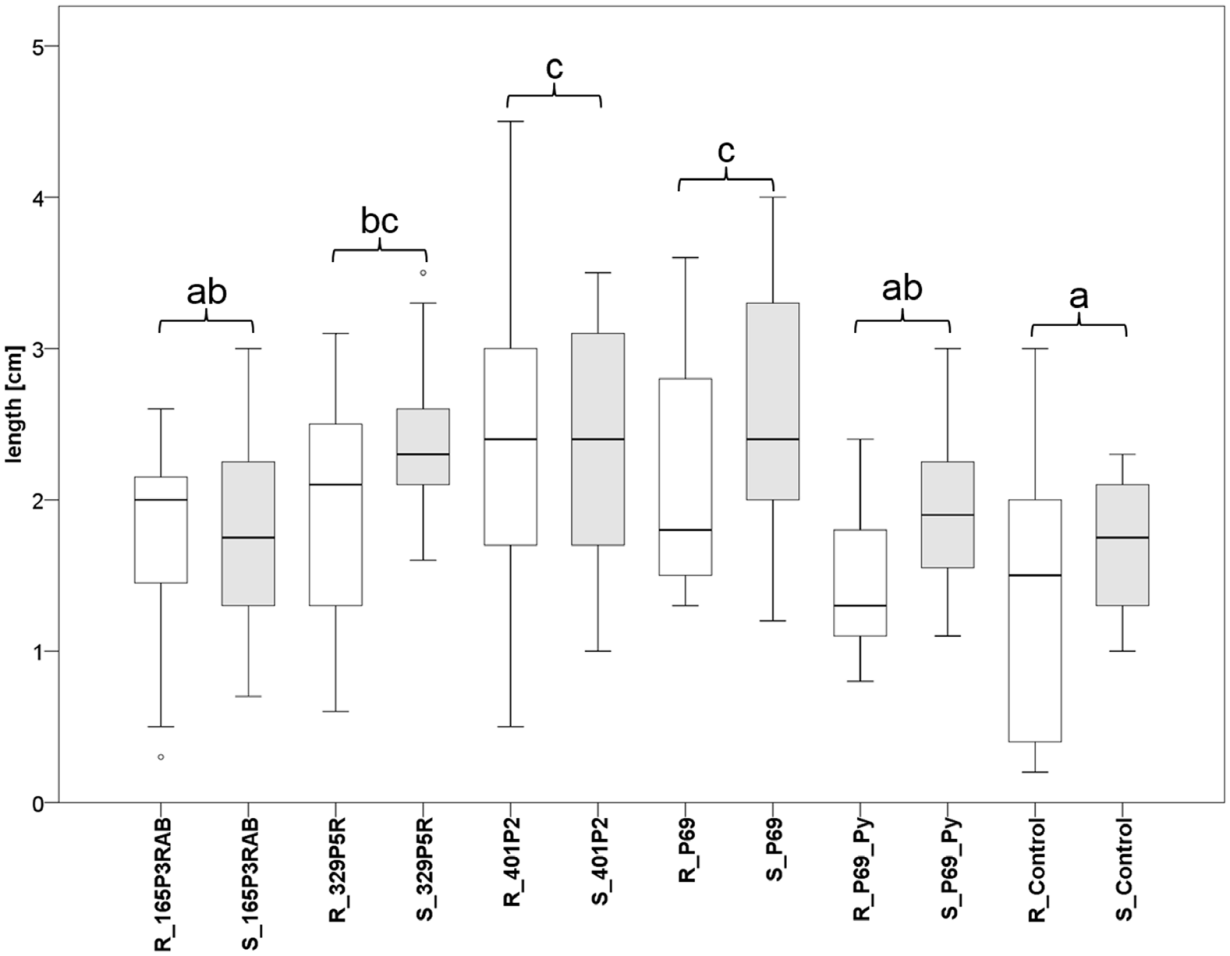
**Greenhouse experiment with *Stenotrophomonas*-primed tomato seeds and two distinctive controls (P69_Py and Control).** Three *Lobaria*-associated isolates (165P3RAB, 329P5R, and 401P2) were utilized together with a plant-associated isolate (P69). Root (R; *n* = 64) and stem (S; *n* = 66) lengths were measured after 2 weeks plant growth under limited water supply. White (root lengths) and gray (stem lengths) plot pairs represent the treatments with specific isolates and complementary controls. Statistical analysis was employed to identify significant (*p* < 0.05) differences in combined root and stem lengths. Different letters were used to differentiate between statistically discriminative groups.

Tomato (*Solanum lycopersicum* L.) seeds were inoculated with three lichen-associated and one plant-associated *Stenotrophomonas* isolate and stress protecting agent as reference (Alavi et al., 2013) to analyze the effect of bacterial inoculants on plants. The primed seeds were grown with limited irrigation for 2 weeks in sterile soil. Two control types were implemented to evaluate growth promotion effects by the inoculants. One control (P69_Py) was supplemented with 60 μL 5-sec-butyl-2,3-dimethylpyrazine per tray during the initial irrigation of *Stenotrophomonas* P69-primed seeds. This heterocyclic compound was found to limit the growth of *Stenotrophomonas* isolates in previous experiments. Correspondingly, the growth of P69-primed samples that were treated with 5-sec-butyl-2,3-dimethylpyrazine (R_P69_Py and S_P69_Py), was similar to non-primed control samples (**Figure 5**). Also, the plant growth was not enhanced by isolate 165P3RAB, which was shown to contain the highest internal spermidine concentrations when compared to both implemented controls. *Stenotrophomonas* isolates 329P5R, 401P2, and P69 enhanced the plant growth significantly when compared to both controls. These isolates were shown to produce low internal spermidine concentrations in previous experiments. Treatments with the lichen-associated isolate 401P2 and the plant-associated isolate P69 resulted in similar plant growth.

## Discussion

The lichen symbiosis was discovered as reservoir for antagonistic bacteria. Interestingly, it was possible to transfer selected isolates from lichens to cultivated plants while maintaining beneficial effects. In addition, we have shown the usability as well as limits of various applied techniques to efficiently screen for specific characteristics and how to reasonably couple classic microbiology with high-end techniques in a comprehensive approach. Starting from a culture collection and dual-culture experiments to screen for active antagonists, the approach was expanded with detailed specification of continuously filtered isolates.

The microbiome involved in the lichen symbiosis is highly diverse (Aschenbrenner et al., 2014) and was identified as bioresource for antagonistic bacteria. *L. pulmonaria* is predominately colonized by *Alphaproteobacteria* (Grube et al., 2015), in particular by various members of *Rhizobiales* (Erlacher et al., 2015b), which harbor many bacterial genera known for a beneficial host-microbe interaction especially with plants. Interestingly, all antagonistic genera identified for lichens – *Stenotrophomonas, Pseudomonas, Burkholderia, Micrococcus, Chryseobacterium, Microbacterium,* and *Paenibacillus* – are well-known from plant studies (Haas and Défago, 2005; Ryan et al., 2009; Rybakova et al., submitted). This is an interesting finding because it shows that these bacteria have the same redundant function independent of the habitat. This observation is underlined by the greenhouse experiments, which have shown that lichen-associated antagonists are active on plants. This also supports the hypothesis that natural ecosystems are interesting reservoirs for biotechnologically relevant bacteria. The present study depicts that cultivable bacterial taxa with lower occurrence on lichens are mainly responsible for the protection against biotic disturbance. A highly diversified bacterial microbiome enhances the available functional repertoire, which might play a crucial role for the stability and longevity of the lichen symbiosis. Comparison of isolate-derived 16S rRNA gene fragments and amplicon-based sequences of abundant antagonists has indicated that a high proportion of *Burkholderia* spp., *Pseudomonas* spp., and *Stenotrophomonas* spp. can be retrieved from lichen symbioses by cultivation experiments on conventional growth media. It was also demonstrated that several isolated antagonists, such as *Bacillus, Paenibacillus*, and *Micrococcus*, were not detectable in the amplicon library but partially in the metagenomic dataset. The most reasonable explanation is that these antagonistic species occur with low abundance within this lichen microbiome and therefore these species might be below the detection limit of the utilized 454 pyrosequencing approach. Other methods with higher coverage might be more suitable to uncover all present bacterial colonizers. Further studies that address this question should preferably subject less multiplexed samples to high-throughput sequencing platforms to obtain a higher read number per sample. This would allow more accurate characterizations of the rare microbial population.

The majority of the antagonistic isolates was assigned to the genus *Stenotrophomonas*. These bacteria have been reported to protect plants against unaffordable conditions like drought and elevated salinity by exudation of protective compounds like spermidine and different osmolytes (Berg et al., 2010; Alavi et al., 2013). Corresponding to this, polyamines which also include spermidine were shown to be involved in plant response to abiotic stress in prior studies (Alcazar et al., 2006; Liu et al., 2007). Environmental strains of *S. maltophilia* and *S. rhizophila* were reported to exert a certain degree of tolerance toward salinity of up to 9% (w/v) NaCl which was correlated with the ability to produce the osmolytes trehalose and glucosylglycerol (Ribbeck-Busch et al., 2005). Even though *Stenotrophomonas*-specific spermidine synthases were not present in the analyzed metagenome, it was evident that utilization of spermidine is widely distributed among various detected organisms. Since lichens presents a habitat that is frequently subjected to drought, the association with bacteria having protective properties appears favorable. However, lichens themselves have mechanisms to account for desiccation, which also includes osmolytes (Green et al., 2011). Moreover, protection mechanisms against oxidative stress-related damage act in a mutual manner among the eukaryotic partners (Kranner et al., 2005). Therefore, we consider stress-protective functions of lichen-associated bacteria to act as an enhancer, which might react more flexibly to local fluctuations of the conditions than their hosts.

Although synthesized and excreted in different amounts, for all selected *Stenotrophomonas* isolates, *in vitro* spermidine production was detected. According to the result, we assume that the function of lichen-associated bacteria includes assistance in the protection against pathogens as well as against damage caused by desiccation. *Stenotrophomonas* is a well-known antagonist of plant-associated origin (Ryan et al., 2009) and connected with a beneficial effect on plant hosts (Berg et al., 1996; Egamberdieva et al., 2011; Alavi et al., 2013). *Stenotrophomonas* strains might also have the same function independent of the habitat. However, in the last two decades, they have received additional attention for opportunistic infections in humans (Berg and Martinez, 2015). It is difficult to identify specific factors of pathogenicity of the virulent isolates but the ability of persistence, resistance and survival – also essential to colonize lichens – allowed a colonization of immunocompromised patients with predisposition. Similar to the plant rhizosphere (Berg et al., 2005), lichens may act as a reservoir for facultative human pathogenic bacteria, or close relatives thereof.

The host-microbiome balance as well as indigenous diversity is essential for functional stability in ecosystems. This balance also depends on the mutual effects among bacteria within the microbiome. In our co-occurrence analyses we found indications for positive and negative correlations among bacterial groups on samples of the same host lichen symbiosis in the same habitat, which is a strong indication of antagonistic and synergistic effect in the lichen habitat. Further analyses are required for clarifying the mechanisms responsible for these effects, as these might involve direct interactions or diffusible metabolites in the system or both. The present results have already shown that lichen symbioses are valuable bioresources to discover bacteria with antagonistic potential and we suggest that a systematic screening of a broader range of lichens may be useful for finding biocontrol solutions that are specifically tailored for ecologically different plant habitats.

## Conflict of Interest Statement

The authors declare that the research was conducted in the absence of any commercial or financial relationships that could be construed as a potential conflict of interest.

## Footnotes

1 http://www.r-project.org

2 http://evolution.genetics.washington.edu/phylip

3 http://tree.bio.ed.ac.uk/software/figtree

4 http://www.ncbi.nlm.nih.gov/genbank
