# Supplementary material for: Analyzing the antagonistic potential of the lichen microbiome against pathogens by bridging metagenomic with culture studies
Source: Front Microbiol. 2015 Jun 22;6:620. doi: 10.3389/fmicb.2015.00620 (PMC4476105; doi:10.3389/fmicb.2015.00620)
Supplement: Supplementary file 1 [file Data_Sheet_1.DOCX]

**Supplementary data**


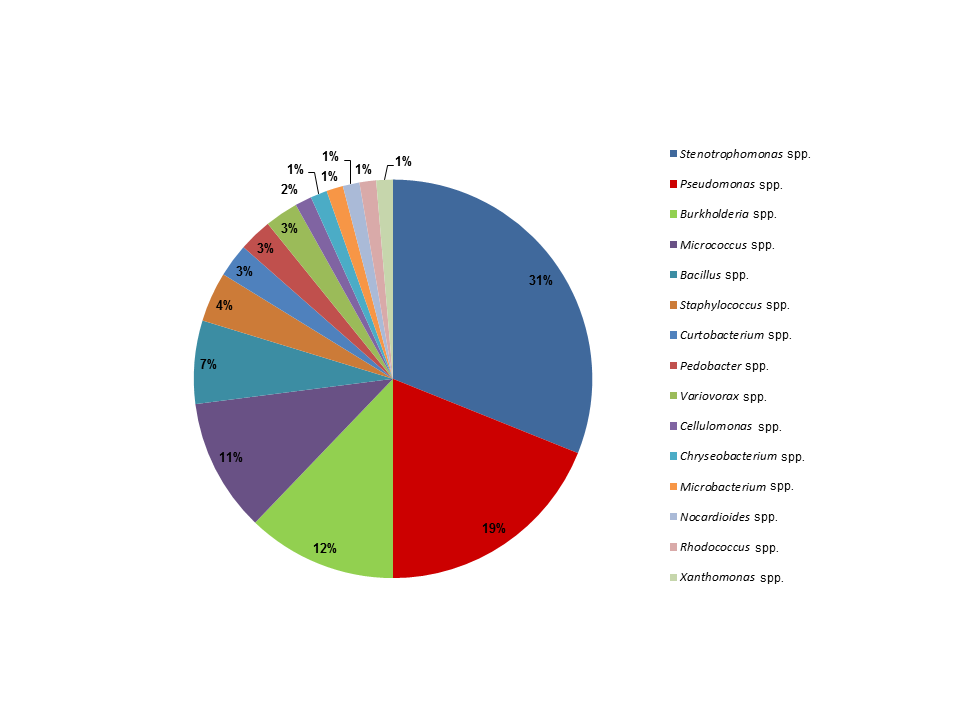


**Figure S1** Visualization of lichen-associated antagonistic bacteria which targeted only one utilized model pathogen. The depicted taxa inhibited growth of either *B. cinerea*, *E. coli*, *S. aureus* or *Rhinocladionella* sp.


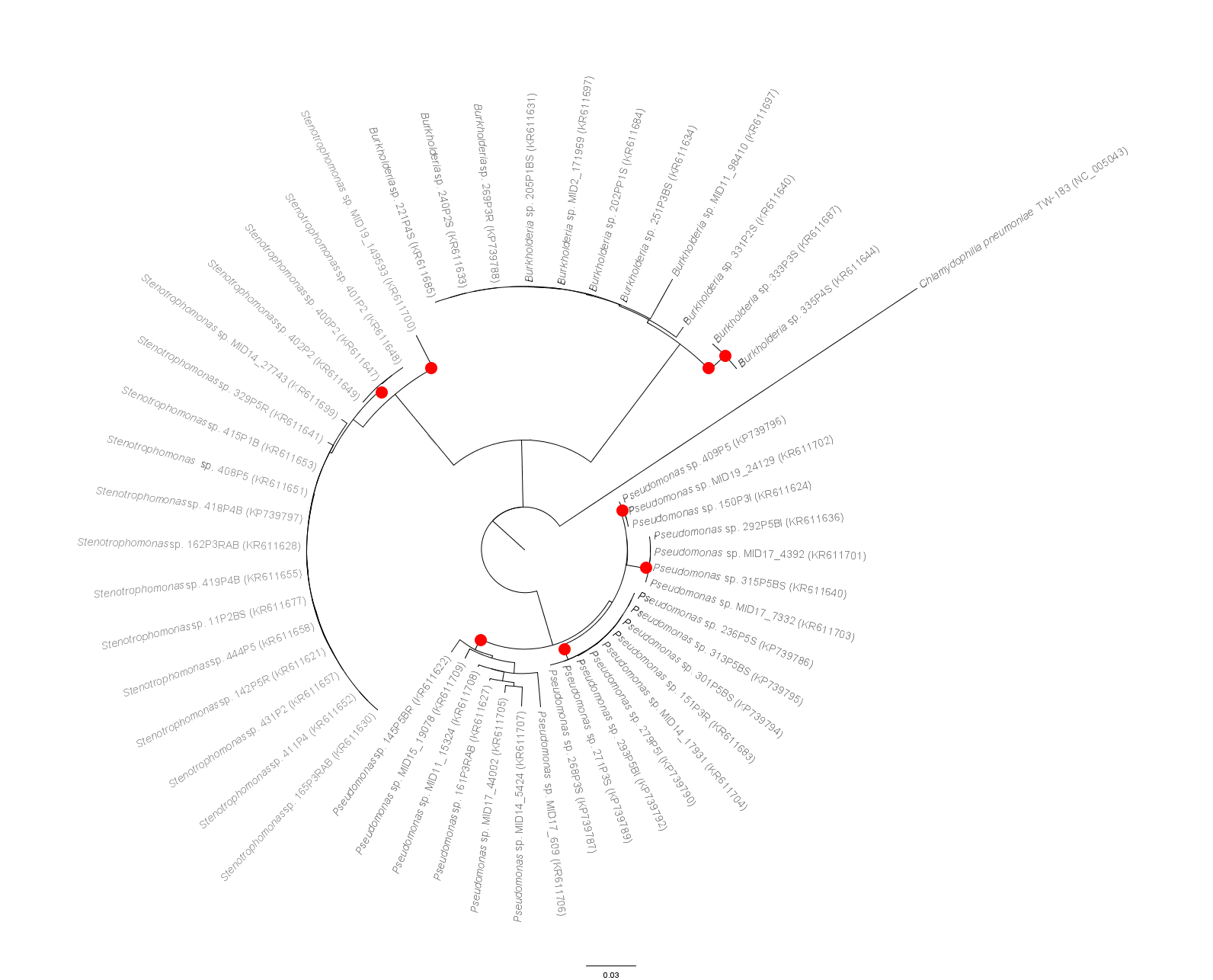


**Figure S2** Neighbor-joining tree based on isolated-derived 16S rRNA gene fragments and specific amplicon sequences from the V4 region. Amplicon-based sequences are labelled with ‘MID’ in the respective sequence names. *Chlamydophila pneumoniae* TW-183 (NC_005043.1) was used as an out-group. Red dots mark nodes with bootstrap values > 70%. Distance bar: 0.03 substitutions per site.


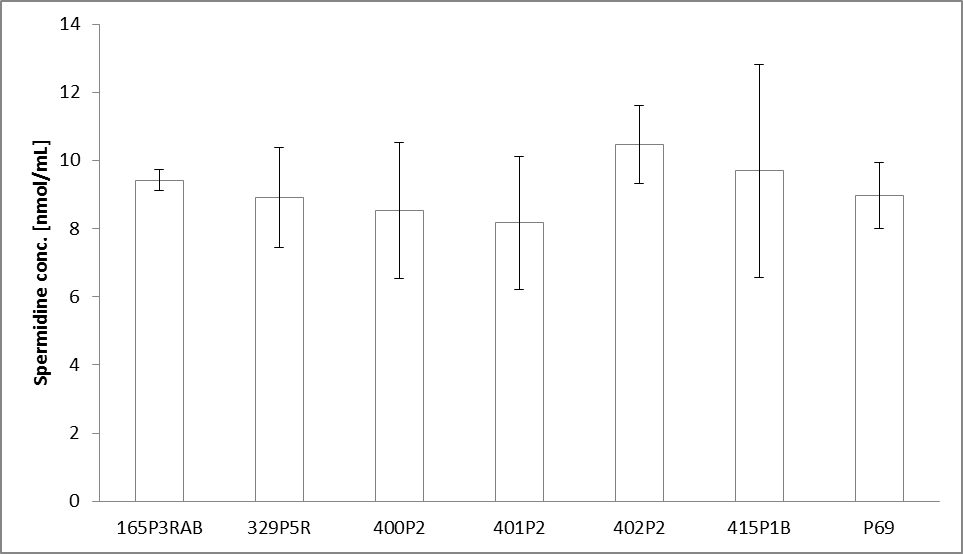


**Figure S3** Analysis of extracellular spermidine concentrations and lichen-associated *Stenotrophomonas* spp. isolates. The isolates were cultivated in fluid cultivation media followed by cell removal and quantification of extracellular spermidine levels. HPLC-MS analysis was utilized to quantify spermidine concentrations. A total of six *Lobaria*-associated isolates was utilized together with one plant-associated isolate (P69) in a comparative approach. ANOVA and Tukey’s HSD post hoc tests did not show significant differences between the samples.


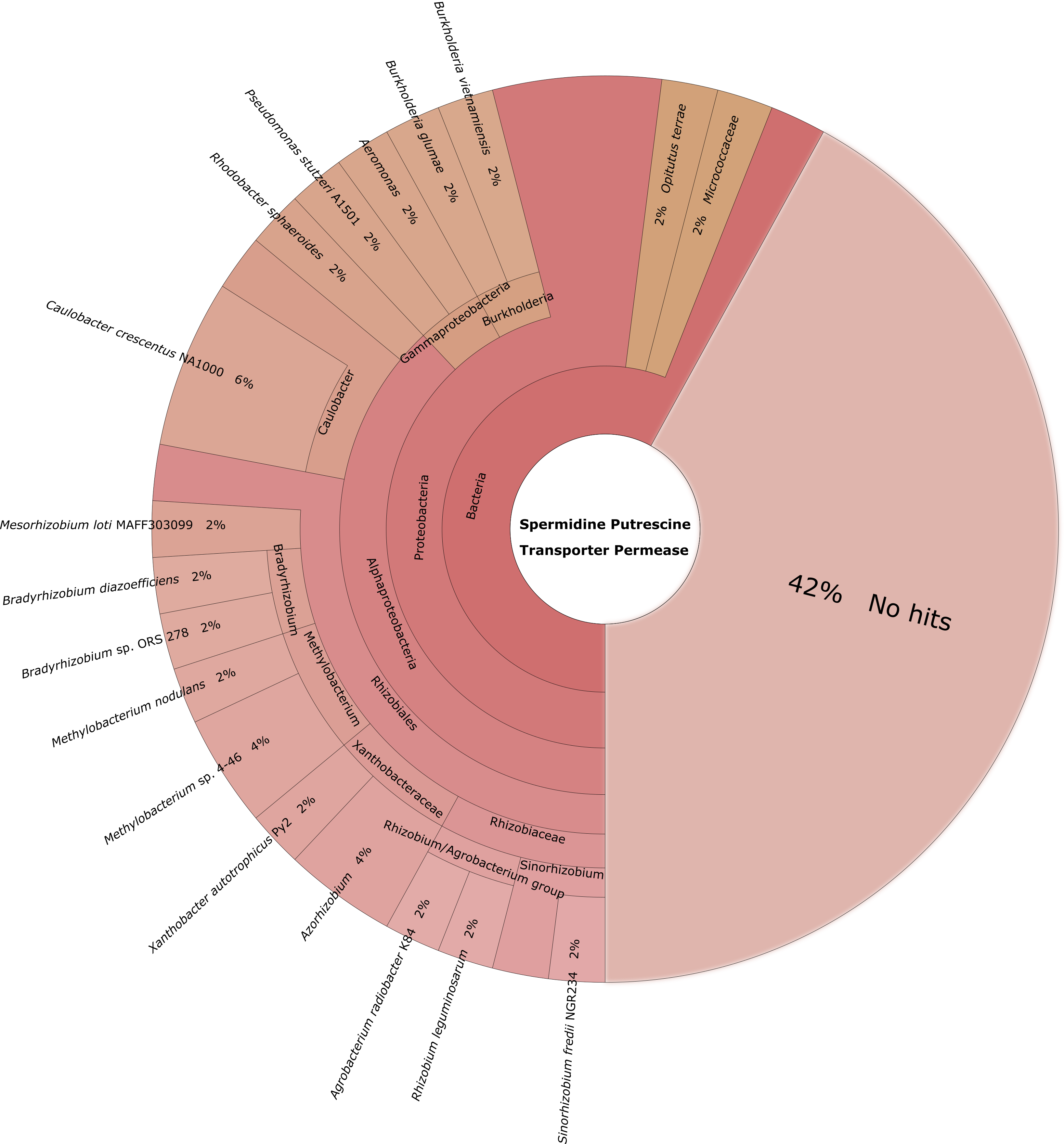


**Figure S4** Visualization of taxonomic assignments of spermidine putrescine transporter permease hits within a *L. pulmonaria* metagenome. The data is visualized in a multi-level chart (http://sourceforge.net/p/krona) with different taxonomic resolutions.
